# RETRACTION: Probiotics Fermentation Technology, a Novel Kefir Product, Ameliorates Cognitive Impairment in Streptozotocin‐Induced Sporadic Alzheimer’s Disease in Mice

**DOI:** 10.1155/omcl/9795039

**Published:** 2026-10-28

**Authors:** Oxidative Medicine and Cellular Longevity

RETRACTION: N. S. El Sayed, E. A. Kandil, and M. H. Ghoneum, “Probiotics Fermentation Technology, a Novel Kefir Product, Ameliorates Cognitive Impairment in Streptozotocin‐Induced Sporadic Alzheimer’s Disease in Mice,” *Oxidative Medicine and Cellular Longevity*, no. 2021 (2021). https://doi.org/10.1155/2021/5525306.

The above article, published online on 8 July 2021 in Wiley Online Library (wileyonlinelibrary.com), has been retracted following the agreement of the Journal’s Associate Editor Angel Catalá; and John Wiley & Sons Ltd.

The retraction has been agreed following an investigation undertaken by the publisher. More specifically, unexpected similarities were identified between the images of the CA3 and CA4 hippocampal regions in the STZ group shown in Figure 11. Further concerns have been identified related to the similarity of the Western Blot bands corresponding to the hippocampal GSK‐3*β* expression in Figure 8 with the bands showing p‐mTOR expression in Figure 4 of [1].

The authors provided a response to the above concerns and provided some of the raw data; however, this was not sufficient to guarantee the integrity of the results. The conclusions of this article can no longer be considered reliable, and it is therefore retracted.

The authors disagree with this retraction.
